# Supplementary material for: Serum DNA methylome of the colorectal cancer serrated pathway enables non‐invasive detection
Source: Mol Oncol. 2024 Jan 10;18(11):2696–713. doi: 10.1002/1878-0261.13573 (PMC11547225; doi:10.1002/1878-0261.13573)
Supplement: Supplementary file 1 — Fig. S1. Global methylation levels of the cfDNA pooled samples. Fig. S2. Results of the differential methylation analysis at probe level. Fig. S3. Distribution of the DMPs obtained from all the pairwise comparisons, relative to CGI and functional genomic locations. Fig. S4. Enrichment of DMPs obtained from all the pairwise comparisons, in relation to CGI annotation and functional genomic regions. Fig. S5. Manhattan plots of differential methylation. Fig. S6. ROC curve analysis and AUC. [file MOL2-18-2696-s001.zip › Full legends Supplementary information.docx]

**Supplementary Figure 1. Global methylation levels of the cfDNA pooled samples.** Boxplot of global cfDNA methylation in NCF, LR-SL, HR-HP and HR-SP pools. Global methylation is expressed as the average methylation of the 734,379 curated probes. The boxplot represents the median (bold line across the box), inter-quartile ranges, and maximum and minimun values (whiskers). HR-HP: high-risk hyperplastic polyp, HR-SP: high-risk serrated polyp, LR-SL: low-risk serrated lesion, NCF: no colorectal findings.

**Supplementary Figure 2.** **Results of the differential methylation analysis at probe level.** Volcano plots showing the -log_10_(*p*-value) versus differences in methylation levels (Δbeta: obtained by subtracting the DNA methylation levels (beta-values) of the two groups involved in each analysis). Significant differentially methylated positions (DMPs) appear highlighted in blue (CpG sites with at least 10% hypomethylation in the most-severe lesion) and orange (CpG sites with at least 10% hypermethylation in the most-severe lesion), and above the red dashed line (p-value < 0.01). HR-HP: high-risk hyperplastic polyp, HR-SP: high-risk serrated polyp, LR-SL: low-risk serrated lesion, NCF: no colorectal findings.

**Supplementary Figure 3**. **Distribution of the DMPs obtained from all the pairwise comparisons, relative to CGI and functional genomic locations.** CGI (CpG island): region of at least 200 bp with a CG content > 50% and an observed-to-expected CpG ratio ≥ 0.6; CGI-shore: sequences 2 kb flanking the CGI, CGI-shelf: sequences 2 kb flanking shore regions, opensea: sequences located outside these regions, promoter regions (5′UTR, TSS200, TSS1500, and first exons), intragenic regions (gene body and 3′UTR), and intergenic regions. TSS200, TSS1500: 200 and 200-1500 bp upstream of the transcription start site, respectively. HR-HP: high-risk hyperplastic polyp, HR-SP: high-risk serrated polyp, LR-SL: low-risk serrated lesion, NCF: no colorectal findings.

**Supplementary Figure 4.** **Enrichment of DMPs obtained from all the pairwise comparisons, in relation to CGI annotation and functional genomic regions.** The colour scale indicates the fold enrichment of all DMPs (grey), hypermethylated (red), and hypomethylated (blue) positions. The bolded numbers indicate annotations that are enriched with respect to the distribution of probes on the MethylationEPIC array (odds-ratio > 1 and one-sided Fisher’s exact test *p*-value < 0.05). CGI (CpG island): region of at least 200 bp with a CG content > 50% and an observed-to-expected CpG ratio ≥ 0.6; CGI-shore: sequences 2 kb flanking the CGI, CGI-shelf: sequences 2 kb flanking shore regions, opensea: sequences located outside these regions. TSS200, TSS1500: 200 and 200-1500 bp upstream of the transcription start site, respectively. HR-HP: high-risk hyperplastic polyp, HR-SP: high-risk serrated polyp, LR-SL: low-risk serrated lesion, NCF: no colorectal findings.

**Supplementary figure 5. Manhattan plots of differential methylation.** Manhattan plots showing –log10(p-value) resulting from differential methylation (left) and absolute differences in the methylation levels (Δbeta-values: obtained by substracting the DNA methylation of NSN from HR-SL) (right) for the 734,739 probes analyzed sorted by chromosome location. Signifficant DMPs appear above the red dashed line (p-value < 0.01 and 10% difference in the methylation levels, respectively).

**Supplementary Figure 6.** **ROC curve analysis and AUC.** ROC curve and AUC for logistic regression models obtained with single or combinations of DMRs for the detection of HR-SL, SAC, HR-HP, or HR-SP, derived by leave-one-out cross-validation in the individual serum samples (n=80). Sensitivity and specificity values for the best cut-offs based on the Youden Index method are highlighted in red. HR-HP: high-risk hyperplastic polyp, HR-SL: high-risk serrated lesion, HR-SP: high-risk serrated polyp, NSN: no serrated neoplasia, SAC: serrated adenocarcinoma.
