# Supplementary material for: Serum DNA methylome of the colorectal cancer serrated pathway enables non‐invasive detection
Source: Mol Oncol. 2024 Jan 10;18(11):2696–713. doi: 10.1002/1878-0261.13573 (PMC11547225; doi:10.1002/1878-0261.13573)
Supplement: Supplementary file 1 — Fig. S1. Global methylation levels of the cfDNA pooled samples. Fig. S2. Results of the differential methylation analysis at probe level. Fig. S3. Distribution of the DMPs obtained from all the pairwise comparisons, relative to CGI and functional genomic locations. Fig. S4. Enrichment of DMPs obtained from all the pairwise comparisons, in relation to CGI annotation and functional genomic regions. Fig. S5. Manhattan plots of differential methylation. Fig. S6. ROC curve analysis and AUC. [file MOL2-18-2696-s001.zip › SupplementaryFigures_final.docx]

**SUPPLEMENTARY FIGURES**

**
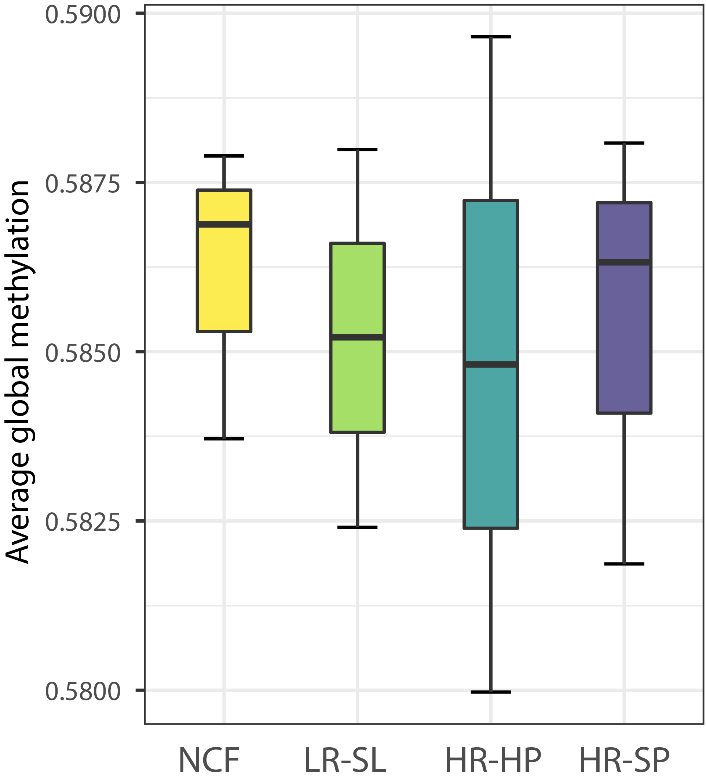
**

**Supplementary Figure 1. Global methylation levels of the cfDNA pooled samples.**

**
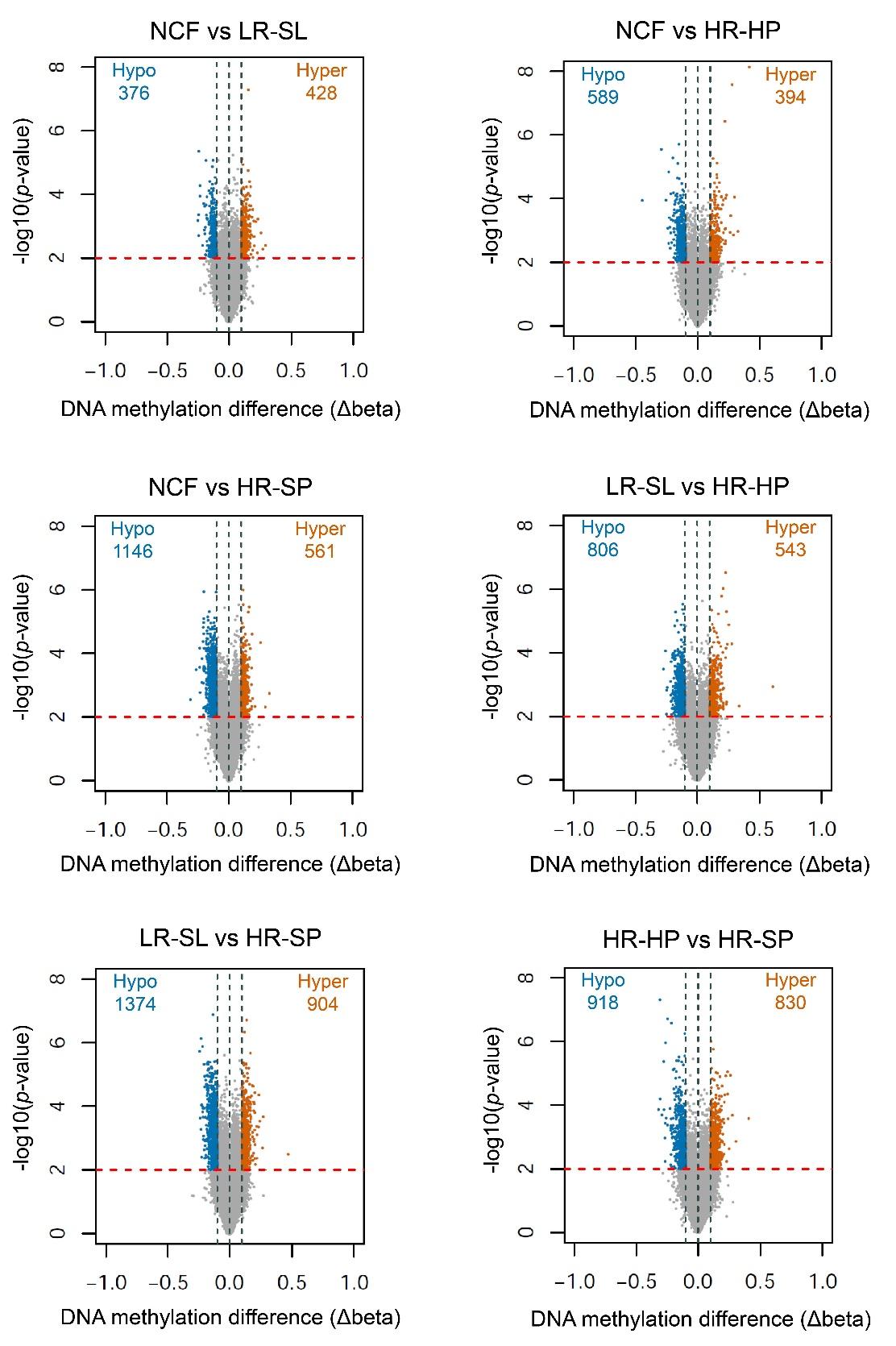
**

**Supplementary Figure 2.** **Results of the differential methylation analysis at probe level.**

**
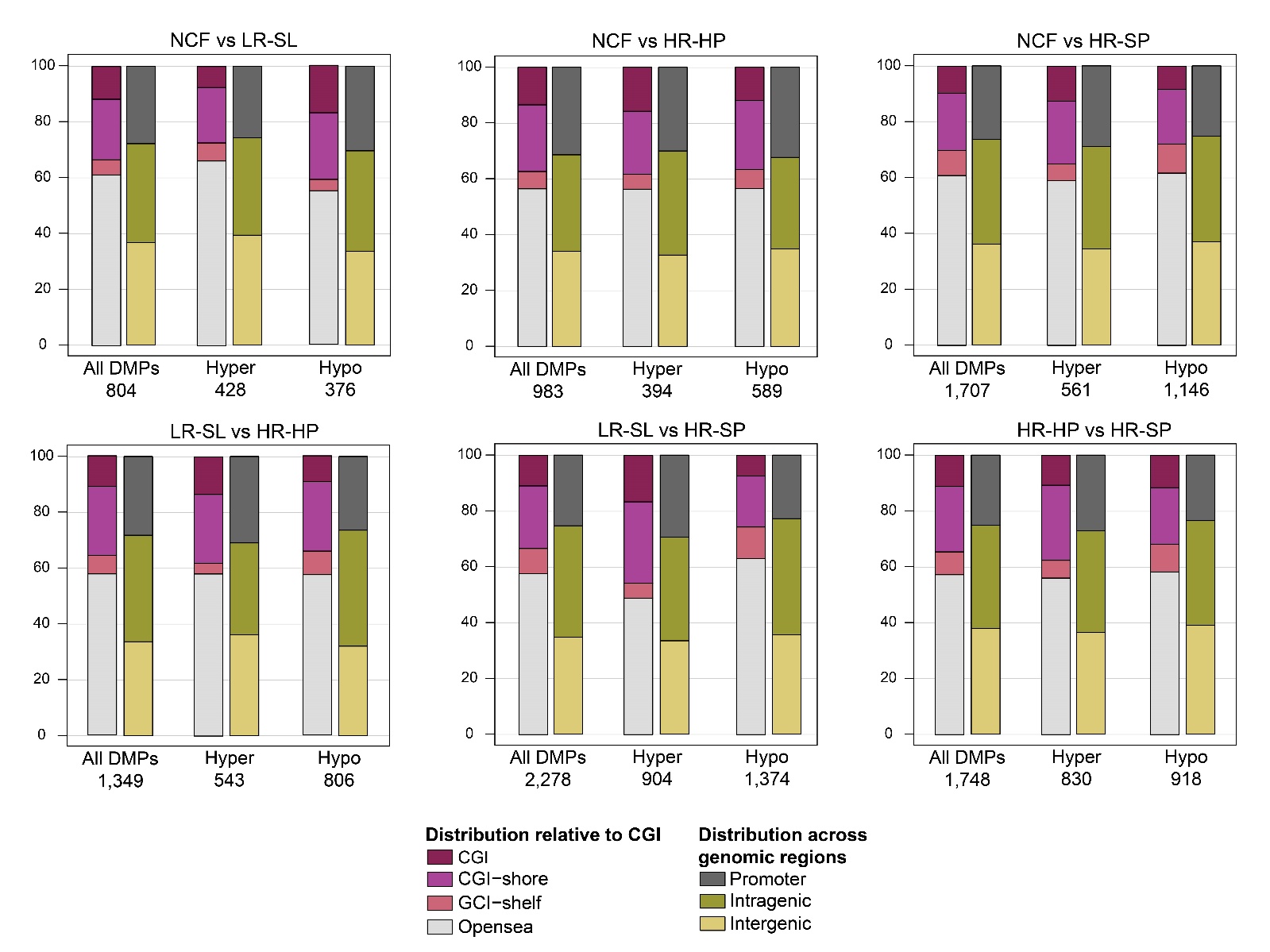
**

**Supplementary Figure 3**. **Distribution of the DMPs obtained from all the pairwise comparisons, relative to CGI and functional genomic locations.**

**
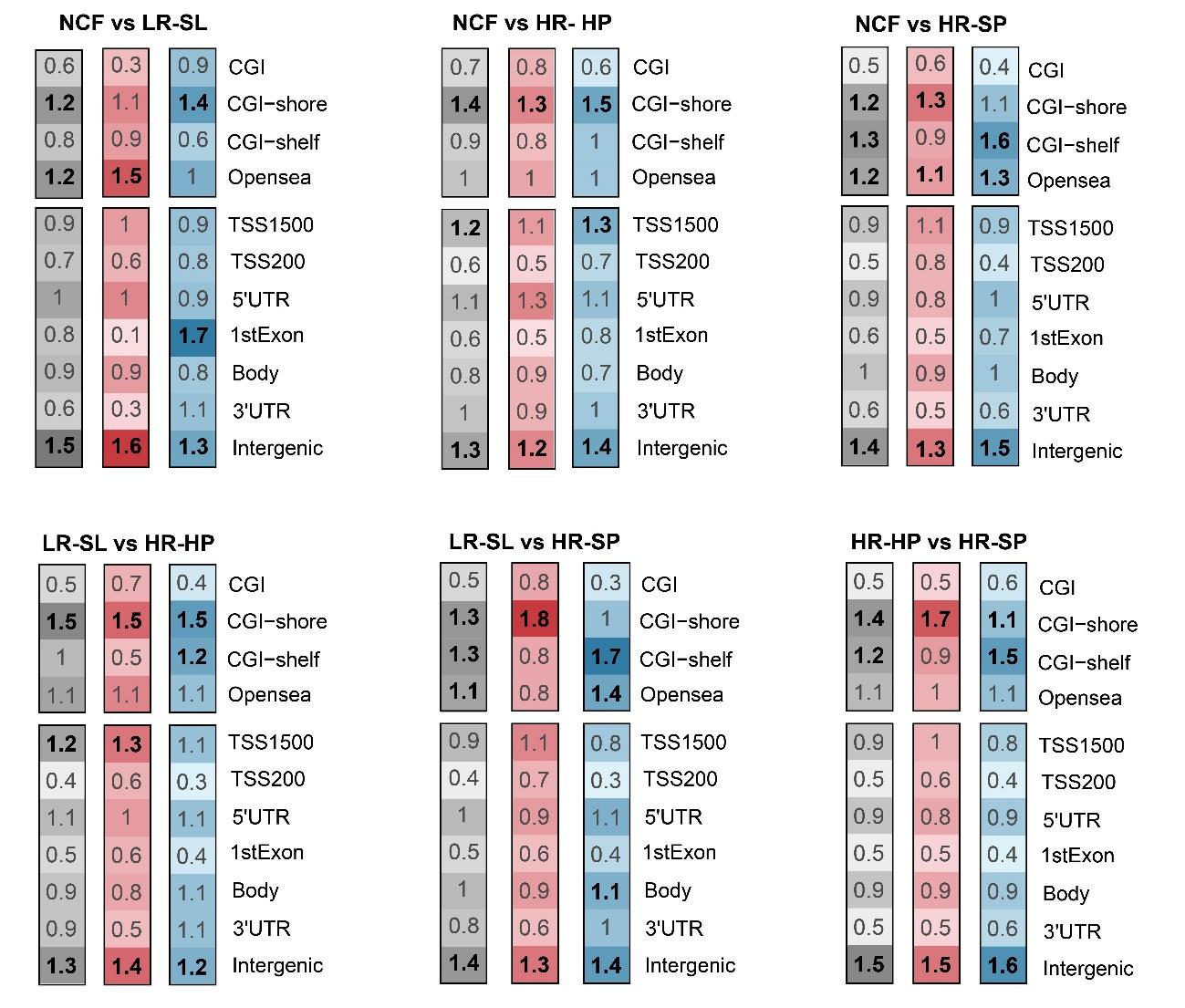
**

**Supplementary Figure 4.** **Enrichment of DMPs obtained from all the pairwise comparisons, in relation to CGI annotation and functional genomic regions.**

**
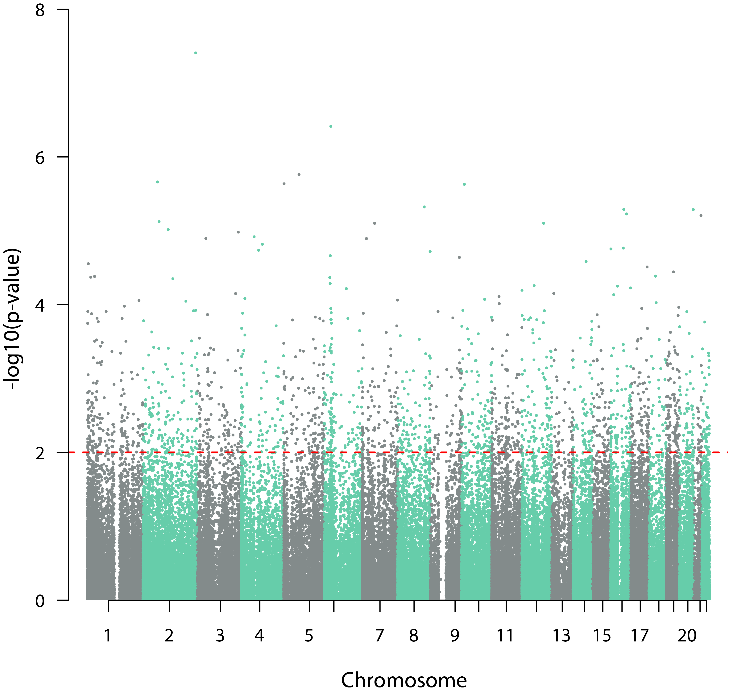

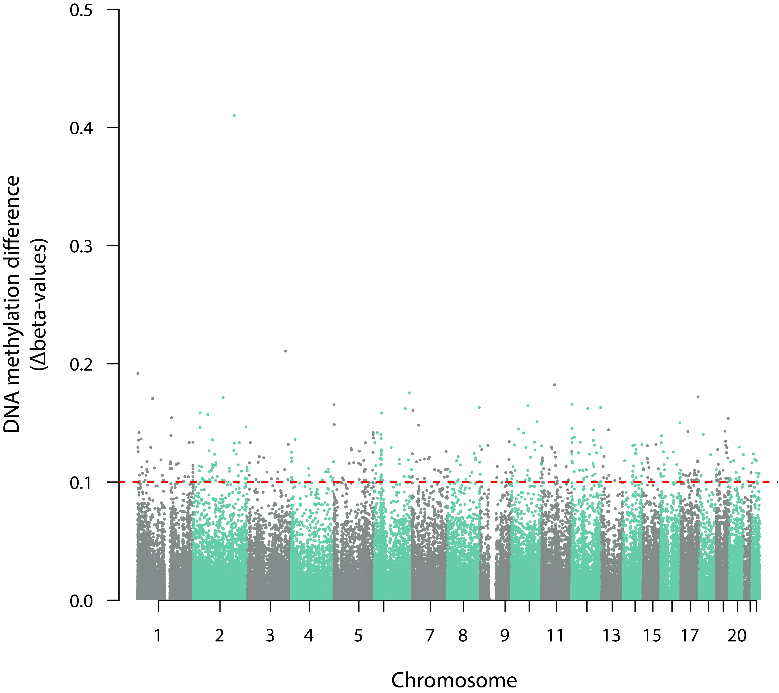
**

**Supplementary figure 5. Manhattan plots of differential methylation.**


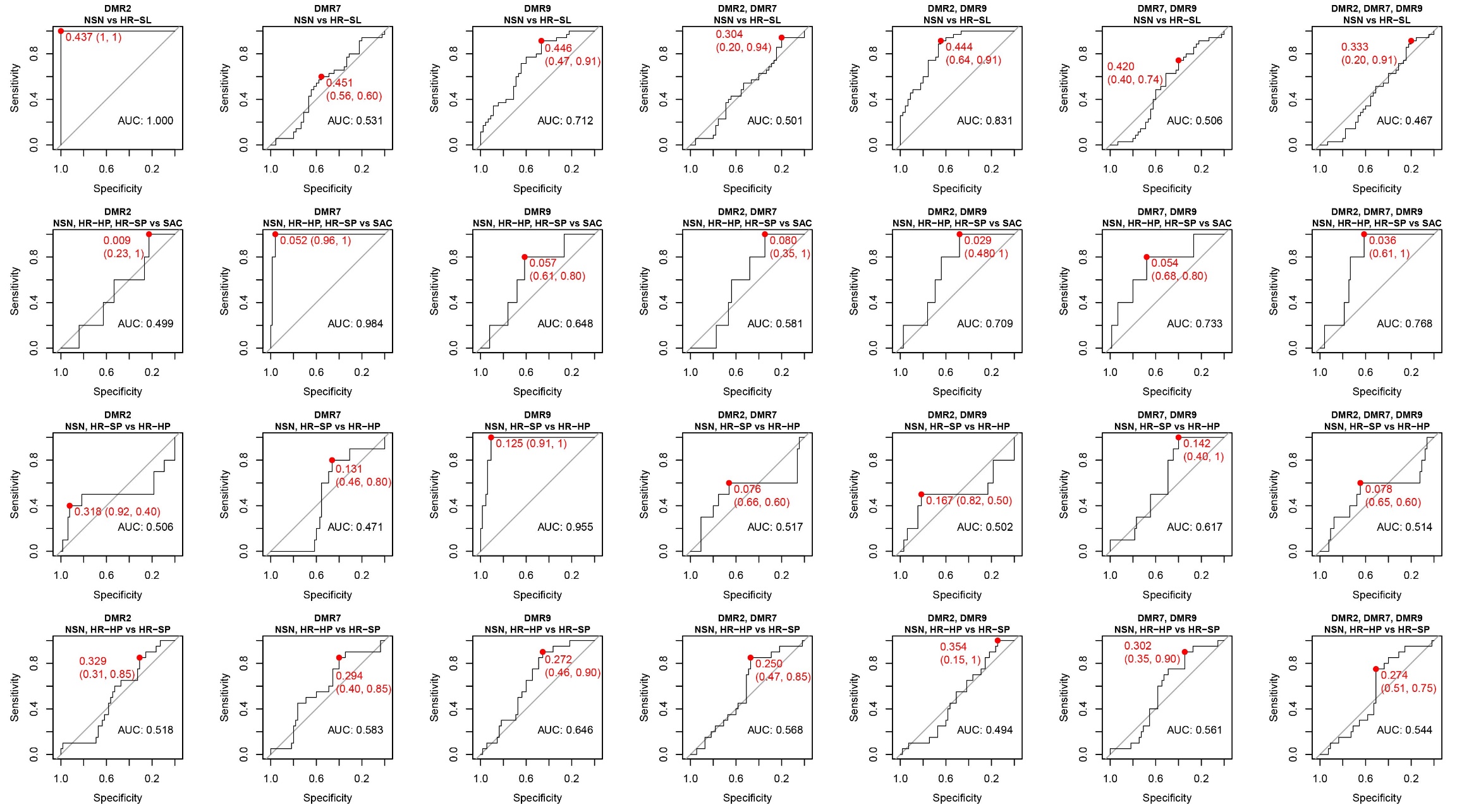


**Supplementary Figure 6.** **ROC curve analysis and AUC.**
