# Supplementary material for: Serum DNA methylome of the colorectal cancer serrated pathway enables non‐invasive detection
Source: Mol Oncol. 2024 Jan 10;18(11):2696–713. doi: 10.1002/1878-0261.13573 (PMC11547225; doi:10.1002/1878-0261.13573)
Supplement: Supplementary file 2 — Table S1. Description of cfDNA pooled samples. Table S2. Primers, probes, and amplicon details for the evaluation of the DMRs in individual serum samples. Table S3. List of the 330 differentially methylated positions (DMPs) between high‐risk serrated lesions (HR‐SL) and no serrated neoplasia (NSN) cfDNA pooled samples. Table S4. List of significantly enriched gene ontology (GO) terms in the hypermethylated and hypomethylated DMPs between high‐risk serrated lesions (HRSL) and no serrated neoplasia (NSN). [file MOL2-18-2696-s002.pdf]

SUPPLEMENTARY TABLES

Supplementary table 1. Description of cfDNA pooled samples.

Pool type	Age median (range)	Total amount DNA (ng)	Pathology description
NCF	61 (53-73)	247.8	Each pool contained 10 individuals with NCF
	61.5 (53-71)	172.8	
	63 (52-72)	123.9	
LR-SL	61.5 (54-74)	269.9	9 Individuals with HP < 10 mm, 1 individual with a SSP < 10 mm
	61 (51-71)	328.0	9 Individuals with HP < 10 mm, 1 individual with a SSP < 10 mm
	61.5 (52-71)	225.6	9 Individuals with HP < 10 mm, 1 individual with a SSP < 10 mm
HR-HP	62.5 (54-68)	259.8	8 Individuals with HP > 10 mm
	61.5 (52-71)	330.2	8 Individuals with HP > 10 mm
HR-SP	61.5 (54-70)	174.4	7 Individuals with dysplastic SSP, 2 individuals with SSP > 10 mm, 1 individual with TSA
	63 (51-71)	298.2	6 Individuals with dysplastic SSP, 2 individuals with SSP > 10 mm, 2 individual with TSA
	62.5 (53-71)	336.0	4 Individuals with dysplastic SSP, 5 individuals with SSP > 10 mm, 1 individual with TSA

NCF: no colorectal findings; HP: hyperplastic polyps, HR-HP: high-risk hyperplastic polyp, HR-SP: high-risk serrated polyp, LR-SL: low-risk serrated lesion, NCF: no colorectal findings, SSL: sessile serrated lesion; TSA: traditional serrated adenoma.

Supplementary table 2. Primers, probes, and amplicon details for the evaluation of the DMRs in individual serum samples. The same pair of primers were used for the pre-amplification and the MS-qPCR. Probes were specific for the fully-methylated sequence. *ACTB* gene was used to normalize for DNA input.

DMR	Primers and probe	Targeted region (GRCh37/hg19)	Length (bp)	CpG sites analyzed
DMR2	Forward: GGGAGTGGGTTTAGTAATGG<br>Reverse: AAACACAATACTCAATACCTAAC<br>Probe: 5´ 6-FAM/TTATAGTTT/ZEN/CGGGTCGGGATTCGTTAG/3IABkFQ	chr12:740220-740319	100	3
DMR7	Forward: TTAGCGYGTTGGATTGATAATTG<br>Reverse: ATACCRCACCCTCCTCTC<br>Probe: 5´ 6-FAM/ACTACGCGC/ZEN/CTCATTCGACAATT/3IABkFQ	chr4:124309-124368	60	3
DMR9	Forward: TGATAGGATYGGGTTTGGGGAAG<br>Reverse: AAATCRCRCTCAACRCAAAAAAAC<br>Probe: 5´ 6-FAM/GTAGAGTTT/ZEN/GAGTATTTCGGATCGCGT/3IABkFQ	chr8:599907-600046	140	3
ACTB	Forward: TCCCTTAAAAATTACAAAAACCACA<br>Reverse: TGGTGATGGAGGAGGTTTAG<br>Probe: 5´ 6-FAM/ACCACCACC/ZEN/CAACACACAATAACAAAAACA/3IABkFQ	chr7:5571748-5571861	114	0

Supplementary table 3. List of the 330 differentially methylated positions (DMPs) between high-risk serrated lesions (HR-SL) and no serrated neoplasia (NSN) cfDNA pooled samples.

**100 hypermethylated DMPs**						
cg02710296	cg07131604	cg06139856	cg14970991	cg24899334	cg21000329	cg18984282
cg14485633	cg25502144	cg24917382	cg16571209	cg02981003	cg10377414	cg00901138
cg15386434	cg13560853	cg18029503	cg04244097	cg14205800	cg17864199	cg25836915
cg08222618	cg09508496	cg23690866	cg27334919	cg07770222	cg10579706	cg02691506
cg11245681	cg15829535	cg24422984	cg14977069	cg06762332	cg20643070	cg02142926
cg16170495	cg02792740	cg09911480	cg11794430	cg10184387	cg17107599	cg07165610
cg11062466	cg02873885	cg14989243	cg25835058	cg22937632	cg01927686	cg15370054
cg00050872	cg24820663	cg17387122	cg07220815	cg05322837	cg22760004	cg12008779
cg05233899	cg27207756	cg16733226	cg09533556	cg01332534	cg02925295	cg11836212
cg08296601	cg15570860	cg17847520	cg08779649	cg14282114	cg24480555	cg00541104
cg16709904	cg14663589	cg09035930	cg20594303	cg20737204	cg08177015	
cg17422692	cg13425294	cg09929369	cg17468267	cg22851864	cg01132407	
cg08767686	cg27089703	cg20016914	cg08369164	cg18583021	cg16955800	
cg10482508	cg19767205	cg01327984	cg02399371	cg01307861	cg22595420	
cg05524354	cg00965110	cg01067216	cg26725559	cg00345425	cg06574229	
**230 hypomethylated DMPs**						
cg10160312	cg25755428	cg06218861	cg10326673	cg14854723	cg11424260	cg00325531
cg26266427	cg27173819	cg16306629	cg08142904	cg09389091	cg15148984	cg06115838
cg02049405	cg10523645	cg10624328	cg08661751	cg02989453	cg23622162	cg08439244
cg12000995	cg27587661	cg04902443	cg03782861	cg04117076	cg06696958	cg14281821
cg20434819	cg21820656	cg08485684	cg23907051	cg21940877	cg00635950	cg26977859
cg16967003	cg13943068	cg08921133	cg23216724	cg14777352	cg01608493	cg03157329
cg07524919	cg01337207	cg15652532	cg00035220	cg14223966	cg03744383	cg24976744
cg05874882	cg03729553	cg03369957	cg02844899	cg01649601	cg19406349	cg20433858
cg19944848	cg15165122	cg16409883	cg26590199	cg21268984	cg08560373	cg12404279
cg08269974	cg14294859	cg19083914	cg10575075	cg09251068	cg21654286	cg07164567
cg17841765	cg00525277	cg00791868	cg05018460	cg03746345	cg04641400	cg04064998
cg10923662	cg07970752	cg00182994	cg15975750	cg15209676	cg22262325	cg23304078
cg23950714	cg18373855	cg24078577	cg07189587	cg15167547	cg15684681	cg17675992
cg19754622	cg06642012	cg10478315	cg25764931	cg05785598	cg09124484	cg08029622
cg22728830	cg07643097	cg05493407	cg15176005	cg11383474	cg10521014	cg03054605
cg19675142	cg20667709	cg01952194	cg11141652	cg13175739	cg20961723	cg01652075
cg05360714	cg00872984	cg26342559	cg27582696	cg04849318	cg25620243	cg04061506
cg06157435	cg23995446	cg21875980	cg18771300	cg06736542	cg20434529	cg05820623
cg26371957	cg02794151	cg20507276	cg20067415	cg01387720	cg20806021	cg20112774
cg03748376	cg27245348	cg19567415	cg08787791	cg01201512	cg01921126	cg22093506
cg07210187	cg13526469	cg10197405	cg00000776	cg14155724	cg01035815	cg23127434
cg23670519	cg15265085	cg12777182	cg15249221	cg06704455	cg06312072	cg04071225
cg08431882	cg18990407	cg04566799	cg25482454	cg26771832	cg08810073	cg15171452
cg05754624	cg05578102	cg00079551	cg08475528	cg23954759	cg19787644	cg01293971
cg16461996	cg09506600	cg25082212	cg18151703	cg20239921	cg02881189	cg23097878
cg20839080	cg25556122	cg11112615	cg24819596	cg00799742	cg00659250	cg03086067
cg09318283	cg05825244	cg01644798	cg18961703	cg26203738	cg07157030	cg26754552
cg01516119	cg01693350	cg14703454	cg12900080	cg05060901	cg26647036	cg07248440
cg07392432	cg15399759	cg02107357	cg10802974	cg02970458	cg20276780	cg01301660
cg03012280	cg19690306	cg00853151	cg14096311	cg03553226	cg23971638	cg09755872
cg05552010	cg04543124	cg20806345	cg09959687	cg08842287	cg10577630	cg16106427
cg11015196	cg08201663	cg21263170	cg26119671	cg08861456	cg22927494	cg03660901
cg23053444	cg00956907	cg08209240	cg18033029	cg07803173	cg02181349	

Supplementary table 4. List of significantly enriched gene ontology (GO) terms in the hypermethylated and hypomethylated DMPs between high-risk serrated lesions (HR-SL) and no serrated neoplasia (NSN).

DMP set	Ontology	GO ID	Name	Over-representation *p*-value	Differentially methylated genes overlapping with the GO term
**Hypermethylated**	BP	GO:0000495	box H/ACA snoRNA 3'-end processing	0.0025	PARN
	BP	GO:0003199	endocardial cushion to mesenchymal transition involved in heart valve formation	0.0034	TWIST1
	BP	GO:0010260	animal organ senescence	0.0036	COMP
	BP	GO:0030042	actin filament depolymerization	0.0046	ADD2, MICAL3
	BP	GO:0032984	protein-containing complex disassembly	0.0009	MRPL28, ADD2, NCKAP5, MICAL3
	BP	GO:0033128	negative regulation of histone phosphorylation	0.0047	TWIST1
	BP	GO:0033979	box H/ACA snoRNA metabolic process	0.0037	PARN
	BP	GO:0034964	box H/ACA snoRNA processing	0.0025	PARN
	BP	GO:0035359	negative regulation of peroxisome proliferator activated receptor signaling pathway	0.0037	TWIST1
	BP	GO:0043624	cellular protein complex disassembly	0.0002	MRPL28, ADD2, NCKAP5, MICAL3
	BP	GO:0051261	protein depolymerization	0.0009	ADD2, NCKAP5, MICAL3
	BP	GO:0090500	endocardial cushion to mesenchymal transition	0.0046	TWIST1
	BP	GO:0090669	telomerase RNA stabilization	0.0046	PARN
	BP	GO:0110008	ncRNA deadenylation	0.0025	PARN
	BP	GO:0140049	regulation of endocardial cushion to mesenchymal transition	0.0010	TWIST1
	BP	GO:0140051	positive regulation of endocardial cushion to mesenchymal transition	0.0010	TWIST1
	BP	GO:2000275	regulation of oxidative phosphorylation uncoupler activity	0.0045	TWIST1
	BP	GO:2000276	negative regulation of oxidative phosphorylation uncoupler activity	0.0023	TWIST1

Supplementary Table 3. (continuation)

DMP set	Ontology	GO ID	Name	Over-representation *p*-value	Differentially methylated genes overlapping with the GO term
**Hypermethylated**	BP	GO:2000802	positive regulation of endocardial cushion to mesenchymal transition involved in heart valve formation	0.0010	TWIST1
	BP	GO:2000800	regulation of endocardial cushion to mesenchymal transition involved in heart valve formation	0.0010	TWIST1
	CC	GO:0070033	synaptobrevin 2-SNAP-25-syntaxin-1a-complexin II complex	0.0050	CPLX2
	MF	GO:0004750	ribulose-phosphate 3-epimerase activity	0.0017	RPEL1
	MF	GO:0016232	HNK-1 sulfotransferase activity	0.0011	CHST10
	MF	GO:0099103	channel activator activity	0.0029	LRRC26
	MF	GO:0099104	potassium channel activator activity	0.0029	LRRC26
**Hypomethylated**	BP	GO:0003312	pancreatic PP cell differentiation	0.0029	NEUROD1
	BP	GO:0003326	pancreatic A cell fate commitment	0.0029	NEUROD1
	BP	GO:0003329	pancreatic PP cell fate commitment	0.0029	NEUROD1
	BP	GO:0014835	myoblast differentiation involved in skeletal muscle regeneration	0.0014	WNT10B
	BP	GO:0045940	positive regulation of steroid metabolic process	0.0025	CES1, FGF1
	BP	GO:0060730	regulation of intestinal epithelial structure maintenance	0.0040	NEUROD1
	BP	GO:0090122	cholesterol ester hydrolysis involved in cholesterol transport	0.0019	CES1
	BP	GO:0090205	positive regulation of cholesterol metabolic process	0.0005	CES1, FGF1
	BP	GO:1903810	L-histidine import across plasma membrane	0.0044	SLC7A1
	MF	GO:0004771	sterol esterase activity	0.0020	CES1, NCEH1
	MF	GO:0004903	growth hormone receptor activity	0.0046	GHR
	MF	GO:0046523	S-methyl-5-thioribose-1-phosphate isomerase activity	0.0020	MRI1

BP: biological process, CC: cellular component, MF: molecular function ontologies.
